# Repeated Isolation of Extended-Spectrum-β-Lactamase-Positive *Escherichia coli* Sequence Types 648 and 131 from Community Wastewater Indicates that Sewage Systems Are Important Sources of Emerging Clones of Antibiotic-Resistant Bacteria

**DOI:** 10.1128/AAC.00823-19

**Published:** 2019-08-23

**Authors:** Erik Paulshus, Kaisa Thorell, Jessica Guzman-Otazo, Enrique Joffre, Patricia Colque, Inger Kühn, Roland Möllby, Henning Sørum, Åsa Sjöling

**Affiliations:** a Department of Food Safety and Infection Biology, Faculty of Veterinary Medicine, Norwegian University of Life Sciences, Oslo, Norway; b Department of Microbiology, Tumor and Cell Biology (MTC), Karolinska Institutet, Solna, Sweden; c Department of Infectious Diseases, Sahlgrenska Academy, University of Gothenburg, Gothenburg, Sweden

**Keywords:** ESBL *E. coli*, Norway, ST131, ST648, antibiotic resistance, antibiotic surveillance, persistence, sewage, wastewater, whole-genome sequencing

## Abstract

Antibiotic resistance in bacteria is an emerging problem globally. Resistant bacteria are found in human and animal microbiota, as well as in the environment. Wastewater receives bacteria from all these sources and thus can provide a measurement of abundance and diversity of antibiotic-resistant bacteria circulating in communities. In this study, water samples were collected from a wastewater pump station in a Norwegian suburban community over a period of 15 months. A total of 45 daily samples were cultured and analyzed for the presence of Escherichia coli. Eighty E. coli-like colonies were collected from each daily sample and then phenotyped and analyzed for antibiotic resistance using the PhenePlate-AREB system. During the sampling period, two unique E. coli phenotypes with resistance to cefotaxime and cefpodoxime indicating carriage of extended-spectrum β-lactamases (ESBL) were observed repeatedly. Whole-genome sequencing of 15 representative isolates from the two phenotypes identified these as two distinct clones belonging to the two globally spread E. coli multilocus sequence types (STs) ST131 and ST648 and carrying *bla*_CTX-M-15_. The number of ESBL-positive E. coli strains in the community wastewater pump station was 314 of 3,123 (10%) analyzed E. coli strains. Of the ESBL-positive isolates, 37% belonged to ST648, and 7% belonged to ST131. Repeated findings of CTX-M-15-positive ST648 and ST131 over time indicate that these STs are resident in the analyzed wastewater systems and/or circulate abundantly in the community.

## Abstract (precis)

Antibiotic resistance in bacteria is an emerging problem globally. Resistant bacteria are found in human and animal microbiota, as well as in the environment. Wastewater receives bacteria from all these sources and thus can provide a measurement of abundance and diversity of antibiotic-resistant bacteria circulating in communities. In this study, water samples were collected from a wastewater pump station in a Norwegian suburban community over a period of 15 months.

## INTRODUCTION

Microbial infectious diseases are a leading cause of morbidity and mortality worldwide. In the past few decades, the incidence of antibiotic resistance among pathogenic bacteria has increased, so surveillance of microbial pathogens and antibiotic-resistant bacteria in clinical settings, communities, and the environment is important (1). Wastewater systems (WWSs) receive a continuous discharge of fecal and urinary waste from human and animal communities (2). Moreover, wastewater treatment plants and sewage are considered reservoirs for bacteria harboring antibiotic resistance genes (ARGs), and might provide a perfect scenario for horizontal gene transfer of ARGs since they represent a unique mixture of antibiotics, disinfectants, metals, resistant bacteria, and fecal microbiota (3). Therefore, WWSs might represent a suitable interface for the surveillance of infectious bacteria and the presence of bacteria carrying ARGs. In addition, if properly collected, WWS samples provide a reliable representation of the large variability of bacteria circulating in large populations connected to the wastewater treatment plant or in individual sewage (4–6). Several studies have suggested an association between the presence of enteric pathogens in WWSs and active disease in the community (2, 7).

Nonpathogenic and commensal Escherichia coli are part of the normal intestinal microbiota of humans and animals. Some strains of E. coli carrying virulence genes are able to cause symptoms and disease in the host. Pathogenic E. coli are classified in two main groups: intestinal pathogenic E. coli and extraintestinal pathogenic E. coli (ExPEC) (8). E. coli strains have been largely recognized for their ability to both harbor and disseminate ARGs to other Enterobacteriaceae when colonizing humans and animals, as well for as their ability to survive in different kinds of environments (9).

Infection by ExPEC strains can produce disease in humans ranging from urinary tract infections (UTIs) to septicemia (8). The emergence of antibiotic resistance, especially E. coli strains producing extended-spectrum β-lactamases (ESBLs), has complicated the medical treatment of ExPEC infections (10). ESBLs consist of a group of enzymes that can hydrolyze broad-spectrum cephalosporins and monobactams but remain susceptible to β-lactamase inhibitors (11, 12). ESBL-producing E. coli sequence type 131 (ST131) is a globally distributed uropathogenic E. coli (UPEC) lineage with higher virulence capacity and antibiotic resistance than other ExPEC clones, which might explain its high occurrence and persistence in urinary and bloodstream infections (10, 13). The global dissemination of E. coli ST131 has been largely related to the pandemic emergence of the CTX-M-15 group of ESBL enzymes (14). The CTX-M-encoding genes probably originate from the chromosomes of various species of the *Kluyvera* genus that were mobilized into enterobacterial plasmids (12). Five different groups of CTX-M and more than 80 different variants have been described. The most commonly found variants are CTX-M-2, CTX-M-3, CTX-M-14, and CTX-M-15 (12). Other ExPEC sequence types, such as ST38, ST405, and ST648, have also been associated with sepsis and UTIs and the global dissemination of different CTX-M variants (10). E. coli ST131 and ST648 producing CTX-M have been reported worldwide, not only in human infections but also in animal samples (13). During the last several decades, a pandemic spread of enzymes belonging to the CTX-M group has been reported. As a consequence, CTX-M-producing enterobacteria, including E. coli, are causing highly antibiotic-resistant infections that are difficult to treat (11, 12).

During a larger study of E. coli isolated from the wastewater of an urban hospital, a suburban community pump station, and a treatment plant in Norway, ESBL carriage was indicated in 7.3% of the isolates by resistance to cefotaxime and cefpodoxime (15). The aim of this study was to further characterize a subpopulation of these ESBL-producing E. coli strains isolated repeatedly from the suburban community wastewater pump station.

## RESULTS

### Two ESBL *E. coli* PhP types were repeatedly found in wastewater from an urban community in Norway.

A total of 3,123 E. coli isolates from the wastewater pump station were analyzed by biochemical fingerprinting (the PhenePlate [PhP] system) and antibiotic resistance screening; 314 of these isolates (10%) exhibited phenotypic resistance to cefotaxime and cefpodoxime, indicating ESBL gene carriage. Of these isolates, several belonged to diverse PhP types that were usually observed in only one sample, and several isolates were also resistant to additional antibiotics. However, two distinct PhP types of ESBL-producing E. coli were repeatedly identified in wastewater samples throughout the sampling period. One PhP type was identified in 115 isolates, and the other was identified in 22 isolates.

During the first and second months of sampling (March and April 2016), nine isolates with the first PhP pattern were isolated that were resistant to ampicillin, cefotaxime, cefpodoxime, trimethoprim, tetracycline, gentamicin, ciprofloxacin, and nalidixic acid. One additional isolate from March 2017 was resistant to all tested antibiotics, i.e., it was also resistant to chloramphenicol, and one isolate from April 2017 was resistant to all tested antibiotics except chloramphenicol and trimethoprim. The remaining 104 isolates of this PhP type had the same resistance pattern to ampicillin, cefotaxime, cefpodoxime, ciprofloxacin, and nalidixic acid. These isolates were found during all months but were particularly abundant in March 2016. After having identified this specific PhP type, we went back to the original data and identified eight additional non-ESBL members of this PhP type. Hence, in total, 3.9% (*n* = 123) of all E. coli isolates belonged to this PhP type, and 93% of these were suspected to be ESBL positive.

The other PhP type was originally isolated as putatively ESBL positive in 22 isolates. Reanalysis identified the same PhP type in a total of 74 isolates from the pump station wastewater; of these, 30% (*n* = 22) were ESBL positive, and half of the ESBL-positive isolates were resistant to all tested antibiotics except chloramphenicol.

### Whole-genome sequencing of the two clonal groups identified them as ST648 and ST131.

**FIG 1:**
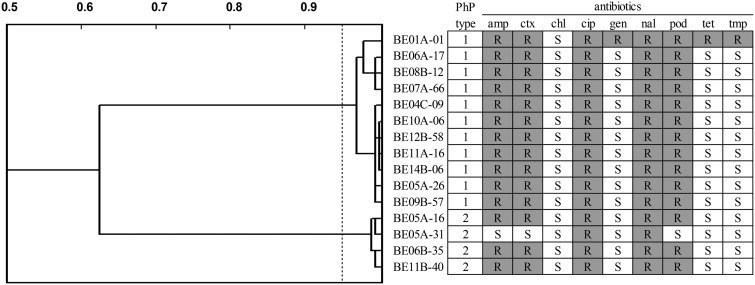
PhP (dendrogram and PhP type) and AREB typing for 15 isolates selected for whole-genome sequencing. Abbreviations: amp, ampicillin; ctx, cefotaxime; chl, chloramphenicol; cip, ciprofloxacin; gen, gentamicin; nal, nalidixic acid; pod, cefpodoxime; tet, tetracycline; tmp, trimethoprim; R, resistant; S, sensitive.

To further study the ESBL clones, a subset of 11 isolates from the first PhP type collected over the whole sampling period, including one multiresistant isolate from March 2016, were cultured, and DNA was extracted. Four isolates from the other PhP type, including one non-ESBL isolate, were also included (Fig. 1). Whole-genome sequencing was performed using an Illumina MiSeq to sequence the genomes of the 15 selected isolates. The genomes were sequenced to a coverage of >100× and assembled. The 15 genomes were analyzed using the CGE MLST finder, and the first PhP type was found to belong to E. coli ST648, while the four isolates of the other PhP type belonged to the uropathogenic E. coli clone ST131 (Fig. 1).

**FIG 2:**
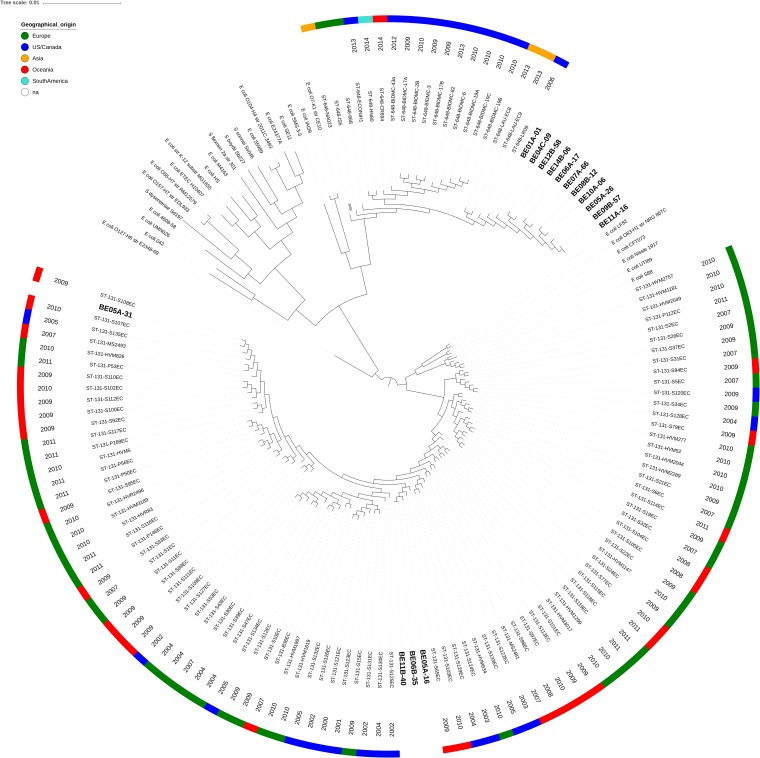
Core genome phylogenetic tree of ST648 and ST131 isolates compared to reference strains. Isolates are labeled by origin and year of isolation. Norwegian ST648 and ST131 isolates are indicated in bold text.

To determine the phylogenetic relationship of the 15 Norwegian isolates to other ST648 and ST131 isolated globally, a reference set of ST648 and ST131 genomes (13) and one ST648 isolate from a river in Bolivia (16) were downloaded from the Sequence Read Archive (SRA). A representative set of E. coli reference genomes (17), spanning the different Clermont E. coli phylogroups (18), was also included in the data set (see Table S1 in the supplemental material). The sequence reads were assembled and annotated using the same settings used for the Norwegian isolates. A phylogenetic tree based on the core genome determined by Roary was constructed (Fig. 2). The phylogenetic tree revealed that the Norwegian ST648 isolates formed a separate monophyletic cluster compared to the global set of isolates. The Norwegian isolates clustered together with an isolate from environmental water in the United States isolated in 2006 and an isolate collected from human feces in Lebanon in 2013. This clade separated from the rest of the ST648 isolates included in the phylogenetic analysis (Fig. 2).

The ST131 isolates clustered at two locations in the phylogenetic tree, indicating that three isolates (ST131-BE05A-16, ST131-BE06B-35, and ST131-BE11B-40) belonged to the previously described ST131 clade A cluster (19), while the fourth, non-ESBL isolate (ST131-BE05A-31), which was resistant only to ciprofloxacin and nalidixic acid (Fig. 1), also belonged to ST131 clade A but clustered closely with isolates S108EC and S107EC isolated in Australia in 2009 and 2010, respectively, that were classified as CTX-M-27 carriers (20). None of the ST131 isolates belonged to the most virulent subtype ST131 clade C H30R.

### *In silico* plasmid analysis revealed several plasmids in the genomes.

The genomes were searched for plasmid content and resistance genes using PlasmidFinder (21) and ResFinder (22), respectively. PlasmidFinder analysis of isolate ST648-BE01A-01, as well as of the four ST131 isolates, detected IncF (Table 1). The IncF plasmids appeared to have been lost in all ST648 isolated after the first group of multiresistant isolates represented by isolate ST648-BE01A-01. Plasmid MLST (pMLST) analyses identified IncF[F1:A1:B1] in ST648-BE01A-01, while IncF[F1:A1:B16] was present in the three ST131 strains that clustered together, and IncF[F1:A2:B20] was present in the non-ESBL ST131-BE05A-31. All ST648 isolates except ST648-BE05A-26 harbored the small plasmid Col8282 and additional smaller plasmids (Table 1). ST131-H30R strains have previously been reported to harbor IncF plasmids, as well as Col156 and Col(MG828) (23). To verify plasmid profiles in all isolates used for the phylogenetic comparison, we analyzed all genomes using PlasmidFinder. IncF, Col8282, Col156, Col(BS512), and Col(MG828) were found in a large number of the isolates. IncN and IncH12 were found in ST648 isolates, while IncF dominated in ST131 (Table S2).

**TABLE 1:** Isolate identification, plasmid profiles and antibiotic resistance genes for 15 selected isolates

Isolate^*a*^	Plasmid(s)	pMLST	Resistance gene(s)
ST648-BE01A-01	Col8282	IncF[F1:A1:B1]	*bla*_CTX-M-15_, *aac(3)IId*, *ARR-3*, *aadA5*, *tet*(34), *aac(6′)-Ib-cr*, *mph*(A), *sul1*, *dfrA17*, *tet*(*B*), *catB3*, *mdf*(A), *bla*_OXA-1_, *ant(3′)*, *bla*_TEM-1B_, *catA1*
ST648-BE04C-09	Col8282		*bla*_CTX-M-15_, *mdf*(A), *tet*(34)
ST648-BE05A-26			*bla*_CTX-M-15_, *mdf*(A), *tet*(34)
ST648-BE06A-17	Col8282		*bla*_CTX-M-15_, *mdf*(A), *tet*(34)
ST648-BE07A-66	Col8282		*bla*_CTX-M-15_, *mdf*(A), *tet*(34)
ST648-BE08B-12	Col8282		*bla*_CTX-M-15_, *mdf*(A), *tet*(34)
ST648-BE09B-57	Col8282		*bla*_CTX-M-15_, *mdf*(A), *tet*(34)
ST648-BE10A-06	Col8282		*bla*_CTX-M-15_, *mdf*(A), *tet*(34)
ST648-BE11A-16	Col8282		*bla*_CTX-M-15_, *mdf*(A), *tet*(34)
ST648-BE12B-58	Col8282		*bla*_CTX-M-15_, *mdf*(A), *tet*(34)
ST648-BE14B-06	Col8282		*bla*_CTX-M-15_, *mdf*(A), *tet*(34)
ST131-BE05A-16	Col(BS512), col156, Col(MG828)	IncF[F1:A1:B16]	*catB3*, *mdf*(A), *aac(6′)-Ib-cr*, *bla*_OXA-1_, *bla*_CTX-M-15_
ST131-BE05A-31	Col8282, col156, Col(MG828)	IncF[F1:A2:B20]	*mdf*(A)
ST131-BE06B-35	Col(BS512), col156, Col(MG828)	IncF[F1:A1:B16]	*catB3*, *mdf*(A), *aac(6′)-Ib-cr*, *bla*_OXA-1_, *bla*_CTX-M-15_
ST131-BE11B-40	Col(BS512), col156, Col(MG828)	IncF[F1:A1:B16]	*catB3*, *mdf*(A), *aac(6′)-Ib-cr*, *bla*_OXA-1_, *bla*_CTX-M-15_

a Isolate names indicate the location (BE), month (01-15, representing each consecutive month from March 2016 to May 2017), and day (A, B, and C) in which they were isolated, as well as colony number.

### The plasmid(s) harboring *bla*_CTX-M-15_ are conserved within, but different between, the ST648 and ST131 clones.

Resistance gene content was analyzed *in silico* using ResFinder. All Norwegian isolates except the non-ESBL ST131-BE05A-31 carried *bla*_CTX-M-15_, which corroborated the phenotypic analysis using the AREB plates and confirmed that these isolates are ESBL carriers. In ST648-BE01A-01, 15 *in silico* resistance genes, in addition to *bla*_CTX-M-15_, were identified using the analysis tools (Table 1). In addition, this isolate was phenotypically resistant to ciprofloxacin and nalidixic acid, indicating additional chromosomal mutations that were not searched for. Since this strain carried an IncF plasmid, apparently lost in the subsequent Norwegian ST648 isolates, these genes might be present on IncF plasmid(s). Manual analyses of contigs revealed that *mph*(A), *sul1*, *aadA5*, *dfrA17*, and *ant(3′)-Ia* resided on the same contig, and *AAR-3*, *catB3*, *bla*_OXA-1_, and *aac(6′)-Ib-cr* were located together on another contig. The *bla*_CTX-M-15_ gene was located in a separate 112,056-bp contig in isolate ST648-BE01A-01. Manual BLAST analysis of the contig revealed that *bla*_CTX-M-15_ is located together with repFIB. The sequence was found to be 99% identical to the 112,210-bp circularized plasmid AnCo1, previously described in E. coli strain 243 isolated from feces of wildlife in Colorado (24), and 99% identical to plasmid pV234-a, a 112,009-bp plasmid isolated from river water in India (25). The contig was also 96% identical to the 109,071-bp plasmid AnCo2, identified in E. coli strain 244 from the same study as AnCo1 (24). AnCo1, pV234-a, and AnCo2 all harbor *bla*_CTX-M-15_. BLAST analysis of the shorter contigs harboring the remaining resistance genes in ST648-BE01A-01 indicated all genes to be plasmid borne. The content of the two contigs containing either (i) *mph*(A), *sul1*, *aadA5*, *dfrA17*, and *ant(3′)-Ia* or (ii) *AAR-3*, *catB3*, *bla*_OXA-1_, and *aac(6′)-Ib-cr* was found on several plasmids in GenBank with conserved gene order and intergenic sequences.

Since *bla*_CTX-M-15_ was found in all ST648 and three of the ST131 isolates, we next analyzed the genetic content of all *bla*_CTX-M-15_ contigs. The genetic location of *bla*_CTX-M-15_ was identical in all ST648 isolates, as well as identical to the location in the previously reported plasmids AnCo1, AnCo2, and pV234-a. Hence, the ST648 isolates harbor a *bla*_CTX-M-15_ plasmid that is evolutionarily conserved and appear to be compatible with the chromosomal background of ST648. Several hypothetical genes, as well as the aerobic cobaltochelatase subunits *cobS* and *cobT*, glutaredoxin, and tRNA genes, flank the *bla*_CTX-M-15_ gene in the plasmid. The ST131 isolates had a smaller contig with a length of 6,169 bp containing the *bla*_CTX-M-15_ gene. The contig had 100% homology to larger plasmids isolated from other E. coli ST131 isolates, including an ST131 O25b:H4, as well as to plasmids from *Klebsiella* spp., *Citrobacter* spp., *Enterobacter* spp., Salmonella enterica, and Shigella sonnei. In the ST131 isolates, *bla*_CTX-M-15_ was located next to a gene encoding the cupin fold-metalloprotein WbuC and surrounded by a Tn*3* family transposase and an IS*Ec9* transposase commonly associated with CTX-M-15.

### The ST648 and ST131 isolates are tolerant to Cu^2+^ and Zn^2+^, and the MIC exceeds the concentrations in wastewater.

The genomes of the 15 sequenced isolates indicated the presence of copper resistance genes, as well as resistance to other heavy metals. We measured the concentrations of copper, zinc, nickel, and chromium ions in all three wastewater sites included in the larger study (15), but the levels were comparable and not higher in the wastewater pump station, and no seasonal variation was observed. The concentrations of copper and zinc in the community wastewater averaged 1.51 μM (extremes, 0.85 to 2.36 μM) and 2.41 μM (extremes, 1.32 to 3.67 μM), respectively, and were about 50-fold higher than those of nickel and chromium. The MICs for the 15 isolates for tolerance toward copper and zinc were determined using agar plates supplemented with copper and zinc sulfate salts. The MICs for the control strains were 16 mM for Cu^2+^ and 4 mM for Zn^2+^, while the MICs for the 15 isolates were similar for all and measured 16 mM for Cu^2+^ and 8 mM for Zn^2+^. The metal tolerance did not increase in the isolates collected later in the study. These results indicate that ST648 and ST131 in this study are tolerant to copper at a level comparable to the control strains and, while slightly more tolerant to zinc than the controls, the levels in the wastewater are probably not high enough to confer any selective pressure that explains the repeated findings of these STs in the wastewater pump station.

### ST131 isolates produce more biofilm than ST648 and display red, dry, and rough biofilm morphotypes.

**FIG 3:**
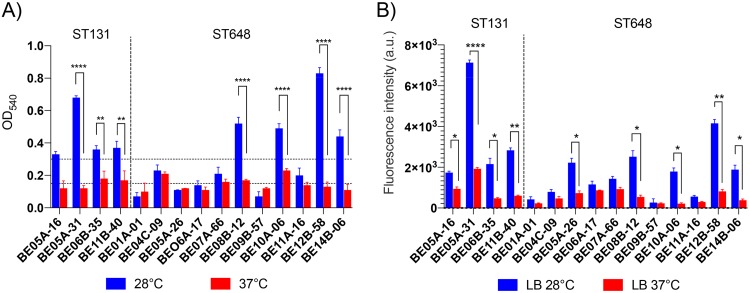
Biofilm formation analysis by E. coli ST131 and ST648. Crystal violet (CV) (A) and FITC-CoA (B) staining of biofilm adherent to polystyrene microplates plates on after incubation for 48 h at 28 or 37°C at the liquid-air interface. For CV staining, retained CV in adherent bacteria is equivalent to the production of biofilm. For FITC-ConA staining, the fluorescence intensity (arbitrary units [a.u.]) represents the EPS production. Horizontal dotted lines indicate low/medium/high biofilm production. Error bars indicate the standard errors of the mean. ****, *P* < 0.0001; ***, *P* < 0.001; **, *P* < 0.01; *, *P* < 0.5.

To assess their ability to form biofilms, the ST131 and ST648 isolates were analyzed by crystal violet staining after incubation at 28 and 37°C. At 28°C, all four ST131 isolates and four of eleven ST648 isolates were classified as high biofilm producers, three ST648 isolates were classified as moderate biofilm producers, and four isolates, including the multiresistant ST648-BE01A-01, were classified as low producers (Fig. 3A). Most isolates showed less biofilm formation at 37°C than at 28°C, and a significant reduction was observed for three ST131 isolates and for four of the ST648 isolates (Fig. 3A).

A similar pattern of biofilm formation was observed in the production of extracellular polymeric substances (EPS) among ST131 and ST648 isolates, with a significant induction of EPS production at 28°C (Fig. 3B). In particular, ST131 isolates showed enhanced EPS production at 28°C compared to ST648 isolates (Fig. 3B; see also Fig. S1 in the supplemental material).

**FIG 4:**
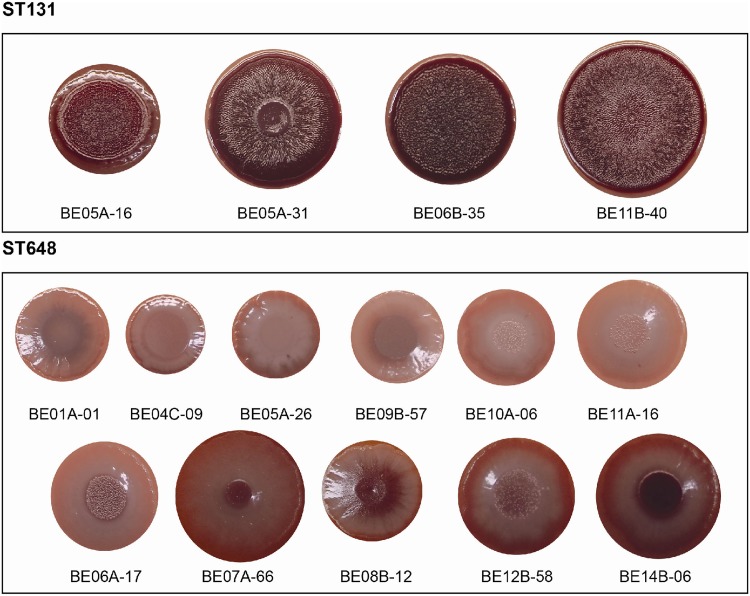
Biofilm phenotypes of Norwegian ST131 and ST648 E. coli isolates. The figure shows rdar morphotype formation of ST131 and ST648 isolates after 48 h of incubation at 28°C on Congo red LB without salt plates.

Evaluation of the production of fimbriae, extracellular polymeric substances, and cellulose by the red, dry, and rough (rdar) morphotype characterization indicated that among all isolates grown at 28°C for 48 h on LB plates without salt (LB_ws_) containing Congo red, the ST131 isolates had a more conserved and fully formed rdar morphotype expressing both cellulose (pdar morphotype), and curli fimbriae (bdar morphotype) (Fig. 4A). On the contrary, the ST648 isolates were smaller and displayed a greater diversity of morphotypes. For example, six of the ST648 isolates (ST648-BE01A-01, ST648-BE04C-09, ST648-BE05A-26, ST648-BE09B-57, ST648-BE10A-06, and ST648-BE11A-16) expressed the smooth-and-white (saw) morphotype without expression of neither cellulose nor curli (Fig. 4B) while the remaining five isolates (ST648-BE06A-17, ST648-BE07A-66, ST648-BE08B-12, ST648-BE12B-58, and ST648-BE14B-06) expressed the bdar morphotype (Fig. 4C). Altogether, our data indicated that 28°C favored biofilm formation, in particular in ST131 isolates.

## DISCUSSION

During the last 10 to 15 years, a global epidemic of ESBL-producing enterobacteria conferred by members of the CTX-M enzymes has emerged (12, 26). CTX-M-encoding genes are probably originally mobilized from the chromosomes of various species of the *Kluyvera* genus to plasmids that are well adapted particularly to E. coli (12). Consequently, ESBL-positive E. coli have increased over time. Carriage of CTX-M enzymes, particularly the CTX-M-15 variant, seems to be associated with specific strains of E. coli. Much of the emergent increase in CTX-M-15 is attributed to E. coli ST131, a pandemic clone that causes ExPEC UTIs and is widely known for worldwide dissemination of multidrug resistance and CTX-M-15 (19). ST131 has emerged as a major human health threat in the last decade, and belongs to a group of so-called high-risk clones, with preference for CTX-M carriage. This group is an important cause of community- and hospital-acquired infections, such as UTIs, surgical-site infections, bloodstream infections, and sepsis, and in addition to ST131 this group includes ST131, ST405, ST410, ST38, ST393, ST69, and ST648 (12, 19, 27, 28). Several of these STs have been identified in animals, birds, and environmental waters and on fresh produce, in addition to humans, indicating a facilitated spread across different niches (28–30). Several STs, including ST648 and ST131, have also been found in environmental water and wastewater (16, 31), supporting the findings of high numbers of ST648 and ST131 in wastewater in this study.

We found that ST131 and ST648 together constituted 44% of all ESBL-positive E. coli in the wastewater pump station. Although the relatively high prevalence of these two sequence types in ESBL-positive isolates is supported by several other studies (32, 33), and ST131 was frequent in a study of clinical, recreational-water, and wastewater samples in the same catchment area as our study (34), one might argue that our findings of ESBL-positive uropathogenic E. coli detected locally in the pump station wastewater is due to fecal matter from one or few persons in the community. To avoid bias caused by grab sampling, wherein one sample reflects a single moment at the time of sampling, continuous sampling over several hours is recommended (35, 36). In this study, a sampling device was used over 24 h for each daily sample (15). Using this sampling technique, the first detection of multiresistant ST648, including isolate ST648-BE01A-01, was during the first and third days of sampling in March and the third day in April. The remaining ST648 isolates with CTX-M-15 and resistance to nalidixic acid and ciprofloxacin were found during all months. The results might suggest that the wastewater is receiving a constant influx of ST648 from the community or, alternatively, that ST648 and ST131 reside locally in the pipes of the wastewater system. Considering the first explanation, the wastewater systems in regular communities may be more relevant to use as sites of monitoring the spread and establishment of globally emerging multidrug-resistant (MDR) bacterial clones related to the intestinal microbiota than so far acknowledged. The focus has primarily been toward establishment in the hospitals and the spread of such clones from hospitals. The alternative explanation indicates that it is possible that wastewater systems constitute a considerable source of resistant bacteria and of resistance genes to be spread into society.

Analysis of metal tolerance in comparison to the levels of copper and zinc in the wastewater did not indicate that the levels of metal ions confer any selective force onto the ST648 and ST131 clones. To analyze their ability to form biofilms, all sequenced isolates were tested using crystal violet analysis and Congo red agar plates. The ST131 isolates were better biofilm producers than the ST648 isolates, which varied in their biofilm-producing capabilities. In accordance with other studies, we could not determine a clear correlation with biofilm production and virulence markers (37).

Uropathogenic E. coli (UPEC) are responsible for 50 and 95% of nosocomial and community UTIs, respectively (10). The intestinal tract in humans has been considered a reservoir of UPEC strains able to infect the urinary tract and to produce severe infections (38, 39). During acute UTIs, infected individuals often have >10^5^ CFU/ml urine (40). Thus, UPEC infections could increase bacterial numbers in urine on an individual and on a community level, which subsequently end up in wastewater treatment plants and the environment. Indeed, UPEC, as well as other types of E. coli, are frequently found in wastewater systems and environmental waters (15, 16, 34, 35).

We found ESBL-positive E. coli ST648 in wastewater, as well as ST131 isolates at lower numbers over several months. ST131 isolates were less frequently ESBL positive compared to ST648. The ST648 clone was first isolated and described in 2008 (41). Although ST648 can cause disease in humans and was first isolated from human urine, the sequence type is also able to cause disease in animals, since it has been isolated from household animals with cystitis and from wild and domesticated animals (42, 43). ST648 has since its discovery been described to be able to carry resistance genes to recent antibiotics, including *bla*_NDM-1_, *bla*_NDM-5_ (44), and *fosA* (45). Our data indicate a higher prevalence of CTX-M-15 in ST648 than in ST131 and ST648 isolates dominated the ESBL-positive E. coli identified in this study. This is corroborated by other studies reporting either similar or higher frequencies of CTX-M-15 carriage in ST648 than in ST131 (32, 43, 46). Interestingly, CTX-M-15 plasmids have been suggested to confer advantage and increase the fitness of its bacterial hosts during infection (47, 48). Further studies are needed to elucidate whether CTX-M-15 plasmids add additional advantageous traits in an ST648 genetic background. However, the conserved CTX-M-15 plasmid identified in the Norwegian ST648 isolates has been found also in the feces of wildlife in the United States (24) and in isolates from river water in India (25). Therefore, the impact of environmental fitness by this plasmid should be determined.

In conclusion, by using PhP plate analysis and confirming observed clones by whole-genome sequencing, we repeatedly identified uropathogenic E. coli belonging to the virulent types ST648 and ST131 and carrying *bla*_CTX-M-15_, as well as several additional resistance genes, in a suburban community wastewater catchment pump station. Additional sampling 2 years later could not identify the PhP types corresponding to these sequence types at the same pump station, which may suggest a transient accumulation of apparently clonal isolates in 2016 and 2017. Monitoring of MDR bacteria in wastewater systems in communities might thus identify emerging clones and/or fluctuations in the bacterial population carrying ARGs and could serve as early warning systems.

## MATERIALS AND METHODS

### Isolation of strains and PhP analysis.

Water samples from a wastewater pump station in a suburban community in Norway were collected over 15 months from March 2016 to May 2017. Sampling was performed as described by Paulshus et al. (15). Briefly, daily samples were collected 3 days in a row each month. Each daily sample was composited of 24 aliquots of 200 ml, collected at hourly intervals for 24 h, using an Isco 3700 full-size portable sampler (Teledyne ISCO, Lincoln, NE). The sampler was rinsed with water between each daily sample and then rinsed with water, cleaned with 0.1 to 1% sodium hypochlorite (Klorin), and bathed in 70% ethanol between monthly sampling occasions. A total of 45 samples were collected over the 15 months. The samples were numbered for the month of collection and labeled A, B, and C for each of the three consecutive sampling days. The daily samples were plated in serial dilutions onto CHROMagar orientation (CHROMagar, Paris, France) agar plates. Up to 80 E. coli-like colonies were collected from each daily sample and subjected to PhenePlate (PhP) typing (PhPlate AB) (5, 35). The PhP system quantifies fermentation patterns for each isolate by assigning numerical values to each carbohydrate source (in 96-plate wells) as a discrete value on a scale from completely negative (25) to completely positive (0), respectively. Each of the 11-cipher profiles per isolate was then compared to all other isolates in the sample and was grouped into PhP types. Isolates within the same PhP type share >97.5% identity in their PhP profiles. Isolates that have identical PhP types are most likely of a common origin or have very similar genomes, i.e., they are clones.

The colonies were simultaneously analyzed for resistance to nine antibiotics with the AREB microplates (5, 35). In total, 3,123 isolates of E. coli were investigated, and 15 isolates were selected to represent isolates from the two observed clonal groups collected over time during different months and with antibiotic resistance patterns of interest. These representative isolates were further characterized in the present study.

### DNA extraction and library preparation.

The 15 selected isolates were grown on LB agar overnight at 37°C. One colony per isolate was picked and used for DNA extraction. The colony was washed with 200 μl of MilliQ water, and bacterial lysis was immediately initiated by treatment with 1 mg/ml lysozyme and proteinase K in lysis buffer overnight. DNA was extracted by using a DNeasy blood and tissue extraction kit (Qiagen), as recommended by the manufacturer, and eluted in 200 μl of MilliQ water. The DNA concentration was measured using Qubit, and 50 ng of DNA was used for library preparation. Sequencing libraries were prepared by using a TruSeq Nano DNA high-throughput library prep kit (Illumina, San Diego, CA) with a mean fragment length of 900 bp. Libraries were sequenced using the MiSeq platform (v.3) chemistry and 2 × 300 bp, generating a coverage of >100× for each isolate.

### Bioinformatics analysis.

The sequence data were processed using the BACTpipe assembly and annotation pipeline v.2.6.1 (49), where reads were assessed for species classification using mash-screen (50), quality trimming and filtering was performed using bbduk (51), and *de novo* assembly and quality assessment was performed using SPAdes (52) within the Shovill pipeline v.1.0.0 (https://github.com/tseemann/shovill); the draft genomes were then annotated using prokka (53), and annotation and assembly statistics were collected using MultiQC (54). Sequence typing and plasmid MLST was performed using CGE pipeline v1.1 (55). After obtaining the sequence typing results, a reference set of ST648 and ST131 genomes was downloaded for comparison, together with a representative set of E. coli reference genomes from the different Clermont groups (17; see also Table S1 in the supplemental material). Sequences downloaded from the Sequence Read Archive (SRA) were assembled and annotated using the same pipeline as described above. Upon annotation, a core genome was constructed using the Roary pangenome pipeline (56), and a phylogenetic tree was computed using FastTree (57). The tree was visualized and annotated using iTol (58).

### Heavy metal concentrations in wastewater.

Concentrations of the heavy metals copper (Cu) and zinc (Zn), as well as nickel (Ni) and chromium (Cr), were determined for all wastewater samples by using Eurofins method NS EN ISO 17294-2 (Eurofins Environment Testing AS, Moss, Norway). The limits of quantification were 2.0 μg/liter for zinc and 0.5 μg/liter for the other heavy metals.

### Metal tolerance and MIC determination.

Isolates were cultured and tested for tolerance to ZnSO_4_⋅7H_2_O and CuSO_4_⋅5H_2_O by the agar dilution MIC determination method for metals (59), with some minor modifications. Briefly, Mueller-Hinton medium was used to prepare series of 2-fold dilutions of ZnSO_4_⋅7H_2_O (from 0.5 to 16 mM) and CuSO_4_⋅5H_2_O (from 0.5 to 32 mM). The pH of the medium was adjusted to 5.5 and 7.0, respectively. Plates were inoculated with spots of 2 μl of bacterial suspensions adjusted to a 0.5 McFarland standard. Plates were incubated for 48 h at 37°C to detect bacterial growth. MICs were established as the minimum concentrations of metal salt where bacterial growth was not present (60). Tolerance to metals was evaluated comparing the MIC values obtained from tested isolates with MIC values from the susceptibility controls. The E. coli strains ATCC 25922 and CV601 were used as controls, and determination tests were conducted in triplicate.

### Crystal violet biofilm assays.

Biofilm assays were performed on microtiter plates as described previously (61). Overnight cultures were diluted 1:100 in fresh LB medium without salt. Aliquots (150 μl) of the diluted samples were incubated in the wells of a flat-bottomed 96-well polystyrene microtiter plate (Corning) for 48 h at 28 or 37°C. Each cell culture (100 μl) was transferred into another flat-bottom 96-well polystyrene microtiter plate and spectrophotometrically measured at 600 nm to assess planktonic bacterial growth. After the planktonic bacteria were discarded from the initial microtiter plate, bacterial cells bound to the wells were gently washed twice with 200 μl of phosphate-buffered saline (PBS), air dried, and then stained with 200 μl of 0.1% (wt/vol) crystal violet for 15 min. After incubation, the stained plates were washed four times with distilled water to remove excess crystal violet, and the stained biofilms were solubilized with 200 μl of 80% ethanol. The absorbance (540 nm) was measured with a SpectraMax i3x spectrophotometer (Molecular Devices). For each experiment, the absorbance of the blank control with crystal violet was subtracted from the experimental sample. Three replicates were used in each experiment, and three independent experiments were performed. Biofilm production was classified as low, medium, or high as previously described (62).

### Semiquantitative analysis of extracellular polymeric substances.

For quantification of extracellular polymeric substances (EPS), assays were performed in Corning 96-well black polystyrene microplates with clear, flat bottoms, as described previously (63). After washing steps following overnight incubation, the wells were treated with fixative (3% paraformaldehyde and 0.25% glutaraldehyde in 0.01 M PBS) for 15 min. The wells were subsequently washed and stained with concanavalin A (ConA) conjugated to fluorescein isothiocyanate (FITC; 25 g/ml) for 15 min at room temperature. Each well was subsequently washed in PBS, and the fluorescence at 488 nm was measured with a SpectraMax i3x spectrophotometer plate reader (Molecular Devices). The analysis was repeated in three independent experiments.

### Biofilm morphotype formation assay.

Portions (5 μl) of an overnight culture were centrifuged, and the pellets were suspended in PBS to an optical density at 600 nm of 5. The concentrated cultures were spotted onto LB without salt (LB_ws_) agar plates supplemented with Congo red (40 μg/ml) and Coomassie brilliant blue (20 μg/ml). Plates were incubated at 28°C for 48 h and photographed.

### Statistical analysis.

Statistical significance for the biofilm analysis was determined using two-way analysis of variance, with a *P* value of ≤0.05 considered significant.

### Data availability.

The sequenced genomes have been deposited at GenBank under accession numbers SPIT00000000, SPIU00000000, SPIV00000000, SPIW00000000, SPIX00000000, SPIY00000000, SPIZ00000000, SPJA00000000, SPJB00000000, SPJC00000000, SPJD00000000, SPJE00000000, SPJF00000000, SPJG00000000, and SPJH00000000.

## Supplementary Material

Supplemental file 1
