# Supplementary material for: Repeated Isolation of Extended-Spectrum-β-Lactamase-Positive Escherichia coli Sequence Types 648 and 131 from Community Wastewater Indicates that Sewage Systems Are Important Sources of Emerging Clones of Antibiotic-Resistant Bacteria
Source: Antimicrob Agents Chemother. 2019 Aug 23;63(9):e00823-19. doi: 10.1128/AAC.00823-19 (PMC6709473; doi:10.1128/AAC.00823-19)
Supplement: Supplemental file 1 [file AAC.00823-19-s0001.pdf]

## Supplementary Files

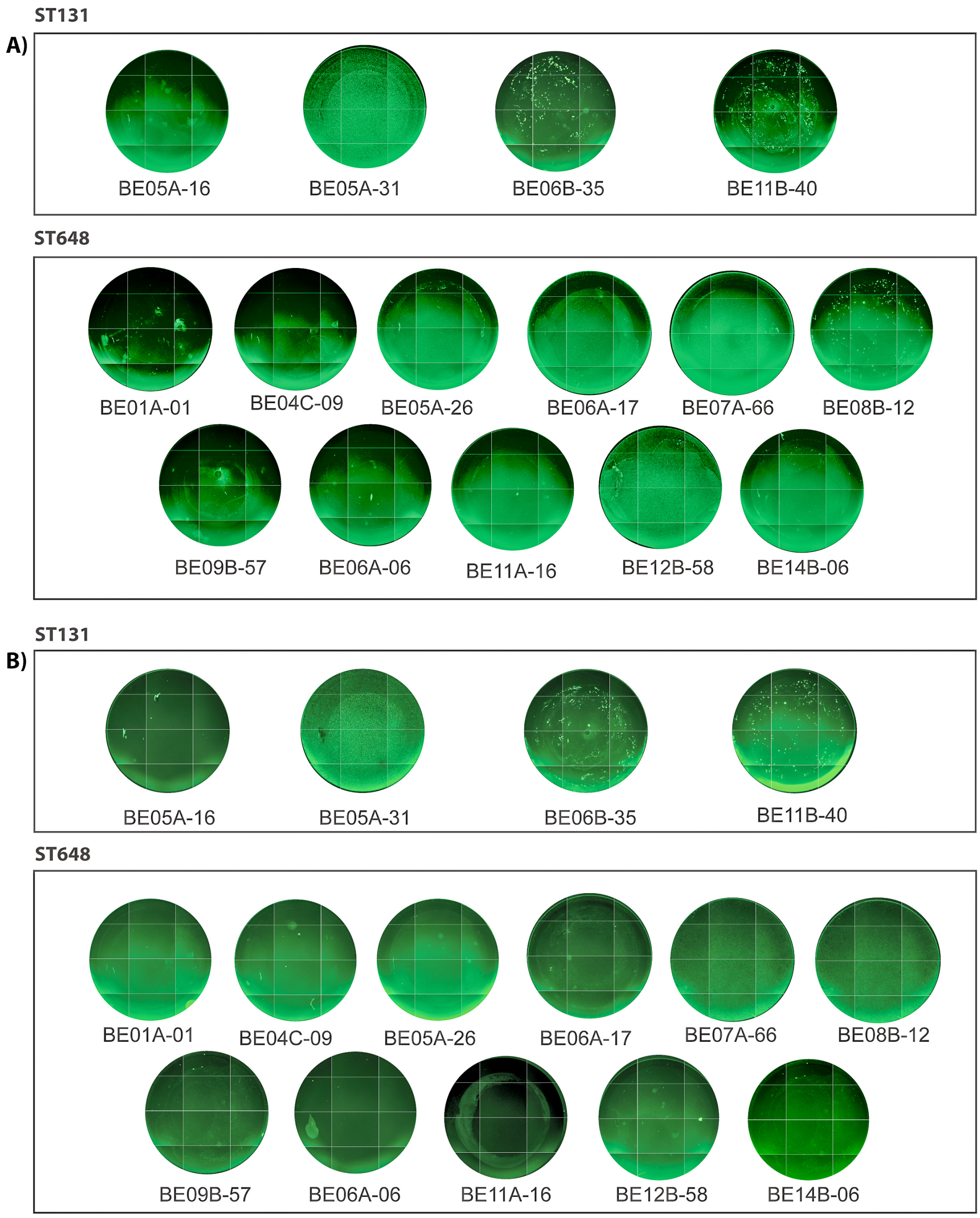


**Supplementary Figure 1. Analysis of exopolysaccharide matrix production** of ST131 and ST648 isolates. Bacterial isolates were grown in 96-well black polystyrene microplate with clear flat bottoms at 28°C (a) and 37°C (b). The isolates were fixed and stained with 25 g/ml FITC-conjugated concanavalin A (green) to assess EPS production. Images are representative of three independent experiments.

**Supplementary Table 1**
Metadata for a set of ST131 and ST648 isolates and reference isolates used for phylogenetic comparison.

| Accession | Strain name | Collection date | Country | host status | isolation source |
|---|---|---|---|---|---|
| ERR161254 | ST-131-B36EC | 2007 | Australia | disease: pyelonephritis | blood |
| ERR161318 | ST-131-HVM1147 | 2010 | Spain | disease: abdominal abscess | Peritoneal fluid |
| ERR161319 | ST-131-HVM1181 | 2010 | Spain | disease: bacteremia | blood |
| ERR161320 | ST-131-HVM1299 | 2010 | Spain | disease: abdominal abscess | Abdominal abscess |
| ERR161321 | ST-131-HVM1619 | 2010 | Spain | disease: surgical wound infection | Surgical wound |
| ERR161322 | ST-131-HVM1997 | 2010 | Spain | disease: pyelonephritis | urine |
| ERR161323 | ST-131-HVM2044 | 2010 | Spain | disease: pyelonephritis | blood |
| ERR161324 | ST-131-HVM2049 | 2010 | Spain | disease: cystitis | urine |
| ERR161325 | ST-131-HVM2289 | 2010 | Spain | disease: cystitis | urine |
| ERR161327 | ST-131-HVM2757 | 2010 | Spain | disease: cystitis | urine |
| ERR161315 | ST-131-HVM277 | 2010 | Spain | disease: cystitis | urine |
| ERR161328 | ST-131-HVM3017 | 2010 | Spain | disease: urinary tract infection | urine |
| ERR161329 | ST-131-HVM3189 | 2010 | Spain | disease: cystitis | urine |
| ERR161306 | ST-131-HVM5 | 2010 | Spain | disease: pyelonephritis | blood |
| ERR161308 | ST-131-HVM52 | 2010 | Spain | disease: pyelonephritis | urine |
| ERR161316 | ST-131-HVM826 | 2010 | Spain | disease: sepsis | blood |
| ERR161317 | ST-131-HVM834 | 2010 | Spain | disease: urinary tract infection | urine |
| ERR161326 | ST-131-HVR2496 | 2010 | Spain | disease: pyelonephritis | blood |
| ERR161311 | ST-131-HVR83 | 2010 | Spain | disease: pyelonephritis | blood |
| ERR161252 | ST-131-MS2481 | 2007 | Australia | disease: bacteremia | blood |
| ERR161253 | ST-131-MS2493 | 2007 | Australia | disease: bacteremia | blood |

| | | | | | |
|---|---|---|---|---|---|
| ERR161312 | ST-131-P112EC | 2011 | Spain | carriage | rectal swab |
| ERR161313 | ST-131-P146EC | 2011 | Spain | carriage | rectal swab |
| ERR161314 | ST-131-P189EC | 2011 | Spain | carriage | rectal swab |
| ERR161307 | ST-131-P50EC | 2011 | Spain | carriage | rectal swab |
| ERR161309 | ST-131-P53EC | 2011 | Spain | carriage | rectal swab |
| ERR161310 | ST-131-P56EC | 2011 | Spain | carriage | rectal swab |
| ERR161263 | ST-131-S100EC | 2009 | Australia | | rectal swab |
| ERR161264 | ST-131-S101EC | 2009 | Australia | | rectal swab |
| ERR161265 | ST-131-S102EC | 2010 | Australia | | urine |
| ERR161266 | ST-131-S103EC | 2010 | Australia | disease: pyuria | urine |
| ERR161267 | ST-131-S104EC | 2008 | Australia | disease: urinary tract infection | urine |
| ERR161268 | ST-131-S105EC | 2008 | Australia | disease: urinary tract infection | urine |
| ERR161270 | ST-131-S107EC | 2010 | Australia | disease: bacteremia | urine |
| ERR161271 | ST-131-S108EC | 2009 | Australia | disease: febrile neutropenia | blood |
| ERR161272 | ST-131-S109EC | 2009 | Australia | carriage | urine |
| ERR161237 | ST-131-S10EC | 2009 | United Kingdom | disease: urinary tract infection | urine |
| ERR161273 | ST-131-S110EC | 2009 | Australia | disease: urinary tract infection | urine |
| ERR161274 | ST-131-S111EC | 2009 | Australia | disease: pyelonephritis | urine |
| ERR161275 | ST-131-S112EC | 2009 | Australia | disease: urinary tract infection | urine |
| ERR161276 | ST-131-S113EC | 2009 | Australia | | rectal swab |
| ERR161277 | ST-131-S114EC | 2011 | Australia | disease: urinary tract infection | urine |
| ERR161278 | ST-131-S115EC | 2011 | Australia | disease: urinary tract infection | urine |
| ERR161279 | ST-131-S116EC | 2011 | United Kingdom | disease: biliary sepsis | |
| ERR161280 | ST-131-S117EC | 2011 | United Kingdom | disease: biliary sepsis | |
| ERR161281 | ST-131-S118EC | 2011 | United Kingdom | disease: urinary tract infection | urine |
| ERR161282 | ST-131-S119EC | 2011 | United Kingdom | disease: bacteremia | blood |
| ERR161238 | ST-131-S11EC | 2009 | United Kingdom | disease: urinary tract infection | urine |
| ERR161283 | ST-131-S120EC | 2009 | Canada | disease: acute cholangitis | blood |

| | | | | | |
|---|---|---|---|---|---|
| ERR161284 | ST-131-S121EC | 2000 | Canada | disease: lower urinary tract infection | urine |
| ERR161285 | ST-131-S122EC | 2003 | Canada | disease: lower urinary tract infection | urine |
| ERR161286 | ST-131-S123EC | 2001 | Canada | disease: lower urinary tract infection | urine |
| ERR161287 | ST-131-S124EC | 2003 | Canada | disease: lower urinary tract infection | urine |
| ERR161288 | ST-131-S125EC | 2002 | Canada | disease: surgical wound infection | Surgical wound |
| ERR161289 | ST-131-S126EC | 2002 | Canada | disease: lower urinary tract infection | urine |
| ERR161290 | ST-131-S127EC | 2002 | Canada | disease: lower urinary tract infection | urine |
| ERR161291 | ST-131-S128EC | 2004 | Canada | disease: sepsis | blood |
| ERR161292 | ST-131-S129EC | 2004 | Canada | disease: lower urinary tract infection | urine |
| ERR161239 | ST-131-S12EC | 2009 | United Kingdom | disease: urinary tract infection | urine |
| ERR161293 | ST-131-S130EC | 2004 | Canada | disease: lower urinary tract infection | urine |
| ERR161294 | ST-131-S131EC | 2002 | Canada | disease: lower urinary tract infection | urine |
| ERR161295 | ST-131-S132EC | 2005 | Canada | disease: lower urinary tract infection | urine |
| ERR161296 | ST-131-S133EC | 2005 | Canada | disease: urosepsis | blood |
| ERR161297 | ST-131-S134EC | 2005 | Canada | disease: lower urinary tract infection | urine |
| ERR161298 | ST-131-S135EC | 2005 | Canada | disease: acute cholangitis | blood |
| ERR161240 | ST-131-S15EC | 2009 | United Kingdom | disease: urinary tract infection | urine |
| ERR161241 | ST-131-S19EC | 2009 | United Kingdom | disease: urinary tract infection | urine |
| ERR161234 | ST-131-S1EC | 2007 | United Kingdom | disease: urinary tract infection | urine |
| ERR161242 | ST-131-S21EC | 2009 | United | disease: | urine |

| | | | | | |
|---|---|---|---|---|---|
| | | | Kingdom | urinary tract infection | |
| ERR161243 | ST-131-S22EC | 2009 | United Kingdom | disease: urinary tract infection | urine |
| ERR161244 | ST-131-S24EC | 2009 | United Kingdom | disease: urinary tract infection | urine |
| ERR161245 | ST-131-S26EC | 2009 | United Kingdom | disease: urinary tract infection | urine |
| ERR161235 | ST-131-S2EC | 2007 | United Kingdom | disease: urinary tract infection | urine |
| ERR161246 | ST-131-S30EC | 2007 | United Kingdom | disease: urinary tract infection | urine |
| ERR161300 | ST-131-S31EC | 2007 | United Kingdom | disease: urinary tract infection | urine |
| ERR161301 | ST-131-S32EC | 2007 | United Kingdom | disease: urinary tract infection | urine |
| ERR161247 | ST-131-S34EC | 2009 | United Kingdom | disease: urinary tract infection | urine |
| ERR161302 | ST-131-S37EC | 2009 | United Kingdom | disease: urinary tract infection | urine |
| ERR161248 | ST-131-S39EC | 2004 | United Kingdom | | |
| ERR161249 | ST-131-S43EC | 2004 | United Kingdom | | |
| ERR161250 | ST-131-S47EC | 2004 | United Kingdom | | |
| ERR161251 | ST-131-S53EC | 2004 | United Kingdom | | |
| ERR161236 | ST-131-S5EC | 2007 | United Kingdom | disease: urinary tract infection | urine |
| ERR161303 | ST-131-S65EC | 2009 | Australia | | urine |
| ERR161299 | ST-131-S6EC | 2007 | United Kingdom | disease: urinary tract infection | urine |
| ERR161304 | ST-131-S77EC | 2010 | Australia | disease: urinary tract infection | urine |
| ERR161305 | ST-131-S79EC | 2009 | Australia | | urine |
| ERR161255 | ST-131-S92EC | 2009 | New Zealand | disease: bacteremia | blood |
| ERR161256 | ST-131-S93EC | 2009 | New Zealand | disease: bacteremia | blood |
| ERR161257 | ST-131-S94EC | 2009 | New Zealand | disease: bacteremia | blood |
| ERR161258 | ST-131-S95EC | 2009 | New Zealand | disease: bacteremia | blood |

| | | | | | |
|---|---|---|---|---|---|
| ERR161259 | ST-131-S96EC | 2010 | New Zealand | disease: bacteremia | blood |
| ERR161260 | ST-131-S97EC | 2010 | New Zealand | disease: bacteremia | blood |
| ERR161261 | ST-131-S98EC | 2008 | Australia | carriage | rectal swab |
| ERR161262 | ST-131-S99EC | 2009 | Australia | carriage | rectal swab |
| SAMN 02471481 | 668 | | Utrecht | | urine |
| SAMN 02138646 | BIDMC-19A | 2010 | Boston, USA | | Broncho-alveolar lavage |
| SAMN 02138647 | BIDMC-19B | 2010 | Boston, USA | | urine |
| SAMN 02138648 | BIDMC-19C | 2010 | Boston, USA | | urine |
| SAMN 02138622 | BIDMC-2B | 2010 | Boston, USA | | blood culture |
| SAMN 02356581 | BIDMC-43a | 2012 | Boston, USA | | blood culture |
| SAMN02138626 | BIDMC-6 | 2010 | Boston, USA | | bile |
| SAMN 02581401 | BIDMC-82 | 2013 | Boston, USA | | urine |
| SAMN 02138640 | BIDMC-17A | 2009 | Boston, USA | | blood culture |
| SAMN02138641 | BIDMC-17B | 2009 | Boston, USA | | Peritoneal fluid |
| SAMN 02138623 | BIDMC-3 | 2009 | Boston, USA | | abdominal incision |
| SAMN 03217331 | CR694 | 2014 | Nambour, Australia | UTI | urine |
| SAMN 02709589 | ECONIH1 | 2013 | USA | | rectal swab |
| | HN80 | | Bolivia | | Environmental River water |
| SAME A3138842 | IS5 | | Vienna, Austria | | unknown |
| SAMN 02470235 | LAU-EC8 | 2013 | Byblos, Lebanon | | gastrointestinal |
| SAMN 02470239 | LAU-EC9 | 2013 | Byblos, Lebanon | | gastrointestinal |
| SAMN 02639609 | LR09 | 2006 | Arkansas, USA | | environmental |
| SAMN 03145047 | NA023 | 2009 | India | | prostitis |
| | E_coli_042 | | | | |
| | E_coli_4608-58 | | | | |
| | E_coli_55989 | | | | |
| | E_coli_CFT073 | | | | |
| | E_coli_E24377A | | | | |
| | E_coli_ETEC_H10407 | | | | |
| | E_coli_HS | | | | |
| | E_coli_IAI39 | | | | |
| | E_coli_LF82 | | | | |
| | E_coli_M4163 | | | | |

| | | | | | |
|---|---|---|---|---|---|
| | E_coli_Nissle_1917 | | | | |
| | E_coli_O104-H4_str_2011C-3493 | | | | |
| | E_coli_O127-H6_str_E2348-69 | | | | |
| | E_coli_O157-H7_str_EDL933 | | | | |
| | E_coli_O55-H7_str_RM12579 | | | | |
| | E_coli_O7-K1_str_CE10 | | | | |
| | E_coli_O83-H1_str_NRG_857C | | | | |
| | E_coli_S88 | | | | |
| | E_coli_SE11 | | | | |
| | E_coli_SMS-3-5 | | | | |
| | E_coli_str_K-12_substr_MG1655 | | | | |
| | E_coli_UMN026 | | | | |
| | E_coli_UTI89 | | | | |
| | S_boydii_Sb227 | | | | |
| | S_dysenteriae_Sd197 | | | | |
| | S_flexneri_2a_str_301 | | | | |
| | S_sonnei_Ss046 | | | | |

**Supplementary Table 2**
PlasmidFinder, ResFinder and VirulenceFinder results on the selected ST131 and ST648 isolates and reference isolates.

| sample name | Resistance | Virulence | plasmids schemed | plasmids unschemed | pMLSTs |
|---|---|---|---|---|---|
| ST-648-NA023 | aac(6')Ib-cr,aadA5,blaCTX-M-15,blaOXA-1,catB3-like,dfrA17,mph(A),strA,strB,sul1,sul2,tet(A) | air,eilA,gad,lpfA | IncFIA, IncFIB(AP001918), IncFII | ColRNAI, Col(MG828) | IncF [F31:A20:B1] |
| ST-648-LR09 | aac(3)-IId-like,aac(6')Ib-cr,aadA5,blaOXA-1,blaTEM-1B,catA1-like,catB3-like,dfrA17,mph(A),strA,strB,sul1,tet(B) | air,eilA,gad,lpfA | IncFII(pRSB107), IncFIA, IncFIB(AP001918) | IncY,ColRNAI, Col(MG828) | IncF[F1:A1:B1] |
| ST-648-LAU-EC9 | aadA2,blaCTX-M-15,catA1-like,dfrA12,erm(B)-like,mph(A),sul1,tet(B) | air,eilA,gad,iha,lpfA,sat | IncFII(pRSB107), IncFIA, IncFIB(AP001918) | Col(BS512), Col8282,Col156, Col(MG828) | IncF [F48:A1:B49] |
| ST-648-LAU-EC8 | aadA2,blaCTX-M-15,catA1-like,dfrA12,erm(B)-like,mph(A),sul1,tet(B) | air,eilA,gad,iha,lpfA,sat | IncFII(pRSB107), IncFIA, IncFIB(AP001918) | Col(BS512), Col8282,Col156, Col(MG828) | IncF [F48:A1:B49] |
| ST-648-IS5 | aac(3)-IId-like,blaCTX-M-15,blaTEM-1B,catA1-like,strA,strB,sul2-like,tet(B) | celb,eilA,gad, lpfA | IncFII(pRSB107), IncFIA, IncFIB(AP001918) | ColRNAI, Col(BS512), Col(MG828), Col156, p0111, IncB/O/K/Z | IncF[F1:A1:B1] |
| ST-648-HN80 | aadA5,blaTEM-1B,dfrA17,erm(B)-like,mph(A),strA-like,strB-like,sul1,tet(B) | air,eilA,gad,lpfA | IncFII(pRSB107), IncFIA, IncFIB(AP001918) | Col(BS512), Col8282,Col156, ColpVC | IncF[F1:A1:B1] |
| ST-648-ECONIH1 | aac(6')-Ib-like,aac(6')Ib-cr-like,aadA1,aadA5,blaCTX-M-15,blaKPC-2,blaOXA-9,blaTEM-1A,dfrA14-like,dfrA17,erm(B)like,mph(A),strA,strB,sul1,sul2 | air,eilA,gad,lpfA | IncFII(pRSB107), IncFIA,IncFIB(AP001918),IncN | | IncN[ST-6], IncF [F1:A1:B1] |
| ST-648-CR694 | aac(6')Ib-cr-like,aadA5,blaCMY-42,blaNDM-5,dfrA17,erm(B)like,mph(A),sul1 | air,eilA,gad,iss, lpfA | IncFII,IncI1,IncFIB(pB171) | ColRNAI, Col(MG828),IncX3,ColE10,IncX4,Col8282 | IncI1[Unknown ST], IncF[F36:A-:B32] |

| | | | | | |
|---|---|---|---|---|---|
| | ,tet(B) | | | | |
| ST-648-BIDMC-82 | aac(6')-IIc, blaCTX-M-14, blaKPC-2,blaSHV-12,blaTEM-1B,dfrA14-like,ere(A)-like,mph(A),sul1,sul2 | air,eilA,gad,iss,lpfA | IncHI2A,IncHI2,IncN | TrfA,ColRNAI, IncX4,IncB/O/K/Z | IncN[ST-6], IncHI2[ST-1] |
| ST-648-BIDMC-6 | aac(6')-IIc,blaKPC-2,blaSHV-12,blaTEM-1B,dfrA14-like,ere(A)-like,mph(A),sul1,sul2 | air,eilA,gad,iss,lpfA | IncHI2A,IncHI2, IncN | TrfA,ColRNAI, IncX4, Col(MG828) | IncN[ST-6], IncHI2[ST-1] |
| ST-648-BIDMC-43a | aac(6')-IIc,blaKPC-2,blaSHV-12,ere(A)-like,sul1,sul2 | air,eilA,gad,iss,lpfA | IncHI2A,IncHI2 | TrfA, Col(MG828), IncX4,ColRNAI | IncHI2[ST-1] |
| ST-648-BIDMC-3 | aac(6')-IIc,blaKPC-2,blaSHV-12,blaTEM-1B,dfrA14-like,ere(A)-like,mph(A),sul1,sul2 | air,eilA,gad,iss,lpfA | IncHI2A,IncHI2, IncN | TrfA,ColRNAI, IncX4, Col(MG828) | IncHI2[ST-1], IncN[ST-6] |
| ST-648-BIDMC-2B | aac(6')-IIc,blaKPC-2,blaSHV-12,blaTEM-1B,dfrA14-like,ere(A)-like,mph(A),sul1,sul2 | air,eilA,gad,iss,lpfA | IncHI2A,IncHI2, IncN | TrfA,ColRNAI, IncX4, Col(MG828) | IncHI2[ST-1], IncN[ST-6] |
| ST-648-BIDMC-19C | aac(6')-IIc,blaKPC-2,blaSHV-12,blaTEM-1B,dfrA14-like,ere(A)-like,mph(A),sul1,sul2 | air,eilA,gad,iss,lpfA | IncHI2A,IncHI2, IncN | TrfA,IncX4, Col(MG828) | IncN[ST-6], IncHI2[ST-1] |
| ST-648-BIDMC-19B | aac(6')-IIc,blaKPC-2,blaSHV-12,dfrA14-like,ere(A)-like,sul1,sul2 | air,eilA,gad,iss,lpfA | IncHI2A,IncHI2, IncN | TrfA,IncX4, Col(MG828) | IncHI2[ST-1], IncN[ST-6] |
| ST-648-BIDMC-19A | aac(6')-IIc,blaKPC-2,blaSHV-12,blaTEM-1B,dfrA14-like,ere(A)-like,mph(A),sul1,sul2 | air,eilA,gad,iss,lpfA | IncHI2A,IncHI2, IncN | TrfA,IncX4, Col(MG828) | IncN[ST-6], IncHI2[ST-1] |
| ST-648-BIDMC-17B | aac(6')-IIc,blaKPC- | air,eilA,gad,iss,lpfA | IncHI2A,IncHI2,IncN | TrfA,ColRNAI, IncX4, | IncN[ST-6], IncHI2[ST-1] |

| | | | | | |
|---|---|---|---|---|---|
| | 2,blaSHV-12,blaTEM-1B,dfrA14-like,ere(A)-like,mph(A),sul1,sul2 | | | Col(MG828) | |
| ST-648-BIDMC-17A | aac(6')-IIc,blaKPC-2,blaLEN12-like,blaSHV-12,blaSHV-12-like,blaTEM-1B,dfrA14-like,ere(A)-like,mph(A),sul1,sul2 | air,eilA,gad,iss,lpfA | IncHI2A,IncHI2,IncN | TrfA,ColRNAI,IncX4,Col(MG828) | IncN[ST-6],IncHI2[ST-1] |
| ST-648-668 | aac(3)-IIa-like,aac(6')Ib-cr,blaCMY-2-like,blaCTX-M-15,blaOXA-1,blaTEM-1B,catB3-like,dfrA25,QnrB2,sul1,tet(A) | air,eilA,lpfA | IncN | Col8282,Col(MG828),ColRNAI,Col156,IncB/O/K/Z | IncN[ST-5] |
| ST-131-S99EC | aadA5,dfrA17,mph(A),sul1,tet(A) | gad,iha,iss,sat | IncFII,IncFIA | Col8282 | IncF[F2:A1:B-] |
| ST-131-S98EC | aac(3)-IId-like,aac(6')Ib-cr-like,aacA4-like,aadA5,blaOXA-1,blaTEM-1B,catA1-like,catB3-like,dfrA17-like,strA,strB,sul1-like,sul2,tet(B) | gad,iha,iss,sat | IncFII(pRSB107),IncFIA,IncFIB(AP001918) | Col(MG828),IncQ1,Col156,ColRNAI | IncF [F48:A1:B49] |
| ST-131-S97EC | aac(3)-IIa-like,aac(6')Ib-cr,aadA5,blaCTX-M-15,blaOXA-1,catB3-like,dfrA17,mph(A),strA,strB-like,sul1,sul2,tet(A)-like | gad,iha,ireA,iss,sat,senB | IncFII(pRSB107),IncFIA,IncFIB(AP001918) | IncY,Col(MG828),Col156,ColRNAI | IncF[F1:A1:B16] |
| ST-131-S96EC | aac(3)-IId-like,blaTEM-1B | gad,iha,iss,sat,senB | IncFII(pRSB107),IncFIA,IncFIB(AP001918) | Col8282,Col156 | IncF[F1:A2:B20] |
| ST-131-S95EC | | gad,iha,nfaE,senB | IncFII(29),IncI1,IncFIB(AP001918) | Col156 | IncI1[Unknown ST],IncF[F29:A-:B10] |
| ST-131-S94EC | blaTEM-1B,tet(A) | gad,iha,iss,sat | IncFIA,IncFII | | IncF[F2:A1:B-] |
| ST-131-S93EC | aadA5,blaTEM-1B,dfrA17,mph(A),sul1 | gad,iha,iss,sat,senB | IncFII(pRSB107),IncFIA,IncFIB(AP001918) | Col156,IncX4,Col(MG828) | IncF[F1:A2:B20] |
| ST-131-S92EC | blaCMY-2 | astA,gad,iha,iroN,iss,mchF,tsh | IncFII(pRSB107),IncI1,IncFIB(AP0 | IncX1,ColRNAI,Col(MG828) | IncI1[ST-12],IncF[F4:A-:B1] |

| | | | | | |
|---|---|---|---|---|---|
| | | | 01918) | | |
| ST-131-S79EC | aadA5,blaCTX-M-15,dfrA17,mph(A),sul1,tet(A)-like | cnf1,gad,iha,iss,sat | IncFII,IncFIA | IncY | IncF[F2:A1:B-] |
| ST-131-S77EC | blaTEM-1B,dfrA14-like,strA-like,strB,sul2,tet(A),tet(B) | gad,iha,iss,nfaE,sat,senB | IncFII,IncFIB(AP001918),IncN | Col156,Col(MG828) | IncF[F51:A-:B10],IncN[ST-3] |
| ST-131-S6EC | blaCTX-M-15,mph(A) | cnf1,gad,iha,iss,sat | IncFII(pRSB107),IncFIB(pB171) | | IncF[F1:A-:B23] |
| ST-131-S65EC | aadA5,blaTEM-1B,dfrA17,mph(A),strA,strB-like,sul1,sul2,tet(A) | gad,iha,nfaE,sat,senB | IncFII(29),IncFIB(AP001918) | Col156 | IncF [F29:A-:B10] |
| ST-131-S5EC | aadA5,blaCMY-23,blaTEM-1B,dfrA17,mph(A),sul1,tet(A) | gad,iha,iss,nfaE,sat | IncFII,IncFIA | Col8282, Col(MG828) | IncF[F2:A1:B-] |
| ST-131-S53EC | aac(6')Ib-cr,aadA5,blaCMY-23,blaCTX-M-15,blaOXA-1,blaTEM-1B,catB3-like,dfrA17,mph(A),sul1,tet(A) | gad,iha,iss,nfaE,sat | IncFII,IncFIA | Col8282, Col(MG828) | IncF[F2:A1:B-] |
| ST-131-S47EC | aac(3)-IIa-like,aac(6')Ib-cr,aadA1-like,aadA5,blaCMY-23,blaCTX-M-15,blaOXA-1,blaOXA-9,blaTEM-1A,catB3-like,dfrA17,mph(A),strA,strB-like,sul1,sul2,tet(A) | cma,gad,iha,ireA,iroN,iss,nfaE,sat,senB | IncFIA, IncFIB(AP001918),IncFII | ColRNAI, Col(MG828), Col156,IncX4, Col8282,ColpVC | IncF [F88*:A1:B16] |
| ST-131-S43EC | aac(6')Ib-cr-like,aacA4-like,aadA1,aadA5,blaCMY-23,blaCTX-M-15,blaOXA-1,blaOXA-9,blaTEM-1C,catB3-like,dfrA17,dfrA5,ere(A),mph(A),strA,strB-like,sul1,sul2,tet(A)-like | gad,iss,nfaE | IncFII,IncN, IncFII(K),IncFIA | IncX4, IncB/O/K/Z, ColpVC, Col(MG828) | IncN[Unknown ST], IncF[F2:A1:B-] |
| ST-131-S39EC | blaTEM-1B,catA1-like,dfrA7,strA-like,strB-like,sul1,sul2,tet(B) | gad,nfaE | IncFII(pRSB107), IncFIA,IncFIB(AP001918), IncFII(pCoo) | | IncF[F1:A1:B1] |

| | | | | | |
|---|---|---|---|---|---|
| ST-131-S37EC | aph(3')-Ia,blaTEM-1B,catA1-like,dfrA14-like,mph(A),strA,strB,sul2,tet(B) | gad,iha,nfaE,sat | IncFII(pRSB107),IncFIA,IncFIB(AP001918),IncFIB(pLF82) | IncQ1 | IncF[F1:A1:B1] |
| ST-131-S34EC | | gad,iha,iss,sat,senB | IncFII,IncFIB(AP001918) | Col156 | IncF[F51:A-:B10] |
| ST-131-S32EC | aph(3')-Ia,blaTEM-1B,tet(B) | gad,iha,senB | IncFII(pRSB107),IncFIA,IncFIB(pB171) | Col156 | IncF[F1:A1:B23] |
| ST-131-S31EC | aadA5,blaCMY-23,blaTEM-1B,dfrA17,mph(A),strA,strB-like,sul1,sul2,tet(A) | gad,iha,iss,nfaE,sat | IncFIA,IncFII | | IncF[F2:A1:B-] |
| ST-131-S30EC | tet(B) | cnf1,gad,senB | IncFII(pRSB107),IncFIA,IncFIB(pB171) | Col156 | IncF[F1:A1:B23] |
| ST-131-S2EC | aadA1,blaTEM-1B,dfrA1,sul1,tet(B) | gad,senB | IncFII(pRSB107),IncFIA,IncFIB(pB171) | Col156 | IncF[F1:A1:B23] |
| ST-131-S26EC | | gad,iha,iss,nfaE,sat,senB | IncFII(29),IncFIB(AP001918) | Col156 | IncF[F29:A-:B10] |
| ST-131-S24EC | | gad,iha,iss,nfaE,sat,senB | IncFII(29),IncFIB(AP001918) | Col156 | IncF[F29:A-:B10] |
| ST-131-S22EC | aadA2,blaTEM-1B-like,catA1-like,dfrA12,mph(A),sul1 | gad,iha,iss,nfaE,sat | IncFII(29),IncFIB(AP001918) | Col(MG828) | IncF[F77:A-:B10] |
| ST-131-S21EC | aac(3)-IIa-like,aadA5,blaCTX-M-15,catB3-like,dfrA17,mph(A)-like,sul1 | gad,iha,iss,sat | IncFIA | ColpVC, Col(MG828) | IncF[F-:A1:B-] |
| ST-131-S1EC | aadA1,blaTEM-1A,dfrA1,sul2,tet(B) | gad,iha,iss,sat,senB | IncFII(pRSB107),IncFIB(AP001918) | IncX1,Col156, IncX4, IncB/O/K/Z | IncF[F4:A-:B10] |
| ST-131-S19EC | aac(6')Ib-cr,blaCTX-M-15,blaOXA-1,blaTEM-1B,catB3-like,dfrA1,strA,strB,sul2,tet(A) | gad,iha,iss,sat | IncFIA,IncFII | IncX1, Col(MG828) | IncF[F2:A1:B-] |
| ST-131-S15EC | aac(3)-IId-like,aadA5,blaCTX-M-14,blaTEM-1B,dfrA17,mph(A),strA,strB-like,sul1,sul2,tet(A) | gad,iha,iss,sat, senB | IncFII(pRSB107), IncFIA, IncFIB(AP001918) | Col156, Col(MG828) | IncF[F1:A2:B20] |
| ST-131-S135EC | aac(6')Ib-cr,aadA5,blaCTX-M-15,blaOXA-1,catB3-like,dfrA17,mph(A),sul1,tet(A) | gad,iha,iss,nfaE,sat | IncFII,IncFIA | p0111 | IncF[F2:A1:B-] |
| ST-131-S134EC | aac(3)-IId-like,aac(6')Ib- | gad,iha,ireA,iss,nfaE,sat | IncFII(pRSB107),IncFIA,IncFIB(AP | Col(BS512),ColRNAI,IncQ1,Col( | IncF[F48:A1:B49] |

| | | | | | |
|---|---|---|---|---|---|
| | cr,aadA5,blaCTX-M-15,blaOXA-1,blaTEM-1B,catA1-like,catB3-like,dfrA17,mph(A),strA,strB,sul1,sul2,tet(B)-like | | 001918) | MG828) | |
| ST-131-S133EC | blaCTX-M-15, tet(A) | gad,iha,iss,sat | IncFII,IncFIA | Col8282, Col(MG828) | IncF[F2:A1:B-] |
| ST-131-S132EC | aac(3)-IIa-like,aac(6')Ib-cr,aadA1,blaCTX-M-15,blaOXA-1,catB3-like,dfrA15,sul1, tet(A) | cma,gad,iha, iroN,iss,sat | IncFIB(AP001918), IncFII(pCoo) | IncX4, Col(MG828) | IncF[F16:A-:B1] |
| ST-131-S131EC | aac(6')Ib-cr,aadA2,aph(3')-Ia,blaCTX-M-15,blaOXA-1,blaTEM-1B,catB3-like,dfrA12,mph(A),sul1,tet(A) | gad,iha,iss,sat | IncFII, IncFIB(AP001918), IncFIA | | IncF [F22:A1:B20] |
| ST-131-S130EC | aac(6')Ib-cr,aadA5,blaCTX-M-15,blaOXA-1,catB3-like,dfrA17,mph(A),sul1,tet(A) | gad,iha,iss,nfaE, sat | IncFII,IncFIA | Col(MG828) | IncF[F2:A1:B-] |
| ST-131-S12EC | aac(3)-IId-like,aac(6')Ib-cr,aadA5,blaCTX-M-15,blaOXA-1,blaTEM-1B,catA1-like,catB3-like,dfrA17,mph(A),strA,strB,sul1,sul2,tet(B)-like | gad,iha,ireA,iss, nfaE,sat | IncFII(pRSB107), IncFIA,IncFIB(AP001918) | Col(BS512),IncY, IncQ1,IncX4,Col(MG828) | IncF[F48:A1:B49] |
| ST-131-S129EC | aadA1, blaSHV-2-like, dfrA1,sul1, tet(A) | gad,iroN,mchF | IncFII,IncI1, IncFIB(AP001918) | ColRNAI, Col(MG828) | IncI1[ST-26], IncF[F24:A:B40] |
| ST-131-S128EC | aac(6')Ib-cr,aadA5,blaCTX-M-15,blaOXA-1,catB3-like,dfrA17,mph(A),sul1,tet(A) | gad,iha,iss,sat | IncFII,IncFIA | Col(BS512), Col156, IncX4 | IncF[F2:A1:B-] |
| ST-131-S127EC | blaCTX-M-15 | gad,iha,iss,sat | IncFII,IncFIA | Col8282, Col(MG828) | IncF[F2:A1:B-] |
| ST-131-S126EC | blaCTX-M-15,blaTEM-1B,dfrA14-like,strA,strB,sul2 | cma,gad,iha,iroN,iss,mchF,sat | IncFIA,IncFIB(AP001918),IncFII | IncY,IncX4 | IncF [F100:A6*:B1] |
| ST-131-S125EC | aac(3)-IId-like,aac(6')Ib- | gad,ireA,iss,nfaE | IncFII(pRSB107), IncFIA,IncFIB(AP | Col(BS512),IncY, IncQ1,IncX4,Col | IncF[F48:A1:B49] |

| | | | | | |
|---|---|---|---|---|---|
| | cr,blaCTX-M-15,blaOXA-1,blaTEM-1B,catA1-like,catB3-like,strA,strB,sul2,tet(B)-like | | 001918) | (MG828) | |
| ST-131-S124EC | aac(3)-IIa-like,aac(6')Ib-cr,blaCTX-M-15,blaOXA-1,blaTEM-1B,catB3-like,tet(A)-like | gad,iha,iss,sat | IncFIA,IncFII | Col(MG828) | IncF[F2:A1:B-] |
| ST-131-S123EC | aac(3)-IIa-like,aac(6')Ib-cr,aadA5,blaCTX-M-15,blaOXA-1,blaTEM-1B,catB3-like,dfrA17,mph(A),sul1,tet(A) | gad,iha,iss,sat | IncFIA, IncFIB(AP001918), IncFII | Col8282 | IncF [F22:A1:B20] |
| ST-131-S122EC | aac(3)-IIa-like,aac(6')Ib-cr,blaCTX-M-15,blaOXA-1,blaTEM-1B,catB3-like,tet(A) | gad,iha,iss,sat | IncFIA,IncFII | Col(MG828) | IncF[F2:A1:B-] |
| ST-131-S121EC | aac(3)-IId-like,aadA5,blaCTX-M-14,blaTEM-1B,dfrA17,mph(A),strA,strB-like,sul1,sul2,tet(A) | gad,iha,nfaE,sat,senB | IncFII(pRSB107), IncFIA,IncFIB(AP001918) | Col156 | IncF[F1:A2:B20] |
| ST-131-S120EC | aac(3)-IIa-like,aac(6')Ib-cr,blaCTX-M-15,blaOXA-1,blaTEM-1C,catB3-like,strA,strB-like,sul2,tet(A) | gad,iha,iss,sat | IncFIA | IncB/O/K/Z, Col8282, ColRNAI, Col156, Col(MG828) | IncF[F-:A1:B-] |
| ST-131-S11EC | aac(3)-IIa-like,aac(6')Ib-cr,aadA5,blaCTX-M-15,blaOXA-1,catB3-like,dfrA17,mph(A),sul1,tet(A) | cnf1,gad,iha,iss,sat | IncFII,IncFIB(AP001918),IncFIA | | IncF[F31:A20:B1] |
| ST-131-S119EC | aac(3)-IIa-like,aac(6')Ib-cr,aadA5,blaCTX-M-15,blaOXA-1,catB3-like,dfrA17,mph(A),sul1,tet(A) | cnf1,gad,iha,iss,sat | IncFII,IncFIB(AP001918),IncFIA | | IncF[F36:A20:B1] |
| ST-131-S118EC | blaTEM-1B-like,strA,strB,sul2 | gad,iha,iss,sat,senB | IncFII(29),IncFIB(AP001918),IncFII(pCoo) | ColRNAI, Col(MG828),Col156,IncX1, Col8282,IncQ1 | IncF [F10:A-:B10] |

| | | | | | |
|---|---|---|---|---|---|
| ST-131-S117EC | aac(3)-IId-like,blaTEM-1B | gad,iha,iss,sat | IncFII(pRSB107),IncFIA | Col8282,Col156 | IncF[F1:A2:B-] |
| ST-131-S116EC | aac(3)-IIa-like,aac(6')Ib-cr,aadA5,blaCTX-M-15,blaOXA-1,blaTEM-1B,catB3-like,dfrA17,sul1 | gad,iha,iss,sat | IncFII,IncFIB(AP001918),IncFIA | ColRNAI, Col(MG828) | IncF [F31:A20:B1] |
| ST-131-S115EC | | gad,iha,iss,sat,senB | IncFII(29),IncFIB(AP001918) | Col156 | IncF [F29:A-:B10] |
| ST-131-S114EC | aac(3)-IIa-like,aac(6')Ib-cr,aadA5,blaCTX-M-15,blaOXA-1,catB3-like,dfrA17,mph(A),sul1,tet(A) | gad,iha,iss,sat | IncFII,IncFIA | Col8282,Col156, IncY, Col(MG828) | IncF[F2:A1:B-] |
| ST-131-S113EC | aadA5,blaCTX-M-3,blaTEM-1B-like,dfrA17,fosA,mph(A),strA,strB-like,sul1,sul2,tet(A) | gad,iha,iss,sat, senB | IncFII(pRSB107), IncFIA, IncFIB(AP001918) | IncX1,Col8282, Col156 | IncF[F1:A2:B20] |
| ST-131-S112EC | aac(3)-IIa-like,aac(6')Ib-cr,aadA2,blaCTX-M-15,blaOXA-1,catB3-like,dfrA12,mph(A),sul1,tet(A) | gad,iha,iss,sat | IncFII,IncFIB(AP001918),IncFIA | Col(BS512), Col8282, Col(MG828) | IncF [F36:A1:B20] |
| ST-131-S111EC | aac(6')Ib-cr,aadA5,blaCTX-M-3,blaTEM-1B-like,dfrA17,fosA,mph(A),strA,strB-like,sul1,sul2,tet(A) | gad,iha,iss,sat | IncFII(pRSB107), IncFIA | IncX1,Col156 | IncF[F1:A2:B-] |
| ST-131-S110EC | aac(6')Ib-cr,aadA5,blaCTX-M-15,blaOXA-1,catB3-like,dfrA17,mph(A),sul1,tet(A) | gad,iha,iss,nfaE, sat | IncFIA,IncFII | Col(MG828) | IncF[F2:A1:B-] |
| ST-131-S10EC | aac(6')Ib-cr,aadA5,blaOXA-1,catB3-like,dfrA17,mph(A),sul1 | gad,iha,iss,sat | IncFIA,IncFII | IncB/O/K/Z, Col156,ColpVC, Col(MG828) | IncF[F2:A1:B-] |
| ST-131-S109EC | aadA5,blaCTX-M-27,dfrA17,mph(A),strA,strB-like,sul1,sul2,tet(A) | gad,iha,iss,sat, senB | IncFII(pRSB107), IncFIA,IncFIB(AP001918) | Col156, Col(MG828) | IncF[F1:A2:B20] |
| ST-131-S108EC | aadA5,blaCTX-M-27,dfrA17,mph(A),strA,strB- | gad,iha,iss,sat, senB | IncFII(pRSB107), IncFIA, IncFIB(AP001918) | Col156, Col(MG828) | IncF[F1:A2:B20] |

| | | | | | |
|---|---|---|---|---|---|
| | like,sul1,sul2,tet(A) | | | | |
| ST-131-S107EC | | NA | IncFIB(K), IncHI1B | | IncHI1 [Unknown ST], IncF[Unknown ST] |
| ST-131-S105EC | | gad,iha,iss,sat, senB | IncFII(29),IncFIB (AP001918) | Col156,IncX4 | IncF[F29:A-:B10] |
| ST-131-S104EC | | gad,iha,iss,sat, senB | IncFII(29),IncFIB (AP001918) | Col156 | IncF[F29:A-:B10] |
| ST-131-S103EC | blaCTX-M-15,mph(A) | gad,iha,iss,nfaE, sat | IncFII(pRSB107), IncFIB(pB171) | Col8282,Col156 | IncF[F1:A-:B23] |
| ST-131-S102EC | aac(6')Ib-cr,blaCTX-M-3,blaTEM-1B-like,fosA | gad,iha,iss,sat, senB | IncFII(pRSB107), IncFIA,IncFIB(AP001918) | IncX1,Col8282, Col156 | IncF[F1:A2:B20] |
| ST-131-S101EC | aac(6')Ib-cr,aadA5,blaCTX-M-15,blaOXA-1,catB3-like,dfrA17,mph(A),sul1,tet(A) | gad,iha,iss,nfaE, sat | IncFII,IncFIA | Col(BS512), Col156 | IncF[F2:A1:B-] |
| ST-131-S100EC | aadA5,blaCTX-M-27,dfrA17,mph(A),strA,strB-like,sul1,sul2,tet(A) | gad,iha,iss,sat, senB | IncFII(pRSB107), IncFIA,IncFIB(AP001918) | Col8282,Col156 | IncF[F1:A2:B20] |
| ST-131-P56EC | aac(6')Ib-cr,aadA5,ARR-3,blaOXA-1,blaTEM-1B,catB3-like,dfrA17,mph(A),strA,strB-like,sul1,sul2,tet(A) | gad,iha,iss,sat, senB | IncFII(pRSB107), IncFIA,IncFIB(AP001918) | Col156, Col(MG828) | IncF[F1:A2:B20] |
| ST-131-P53EC | aac(6')Ib-cr,aadA5,blaCTX-M-15,blaOXA-1,blaTEM-1A-like,catB3-like,dfrA17,mph(A),strA,strB-like,sul1,sul2,tet(A) | gad,iha,iss,sat, senB | IncFII(pRSB107), IncFIA,IncFIB(AP001918) | Col(MG828), Col156,IncX4, ColRNAI | IncF[F4:A2:B20] |
| ST-131-P50EC | blaTEM-1B | celb,gad,iss,sat | IncFII(pRSB107), IncFIA,IncFIB(AP001918) | Col(MG828), Col(pWES),ColRNAI,Col156, IncX1,Col8282, IncB/O/K/Z | IncF[F1:A2:B20] |
| ST-131-P189EC | aac(3)-IId-like,aadA5,blaTEM-1B,dfrA17,sul1 | gad,iha,iss,sat, senB | IncFIA,IncFIB(AP001918) | Col156, Col(MG828) | IncF[F-:A2:B20] |
| ST-131-P146EC | aac(3)-IId-like,aadA5,blaTEM-1B-like,dfrA17,strA, strB-like,sul2 | gad,iha,iss,sat, senB | IncFII(pRSB107), IncFIA,IncFIB(AP001918),IncFII | Col(BS512), Col(MG828), IncB/O/K/Z, ColRNAI | IncF [F1:A2:B20*] |
| ST-131-P112EC | | astA,gad,ireA,iroN,iss,mchF,vat | IncFII,IncFIB(AP001918) | ColRNAI | IncF[F24:A-:B1*] |

| | | | | | |
|---|---|---|---|---|---|
| ST-131-MS2493 | aadA5,blaTEM-1B,dfrA17,mph(A),strA,strB-like,sul1,sul2,tet(A) | gad,iha,iss,sat,senB | IncFII(pRSB107),IncFIA,IncFIB(AP001918) | Col156 | IncF[F1:A2:B20] |
| ST-131-MS2481 | aadA5,blaTEM-1B,dfrA17,mph(A),sul1,tet(A) | gad,iha,ireA,iss,sat | IncFIA,IncFIB(AP001918),IncFII | Col8282, Col(MG828) | IncF[ F22:A1:B20] |
| ST-131-HVR83 | aadA5,blaTEM-1B-like,dfrA17-like,mph(A),strA,strB-like,sul1,sul2,tet(A) | gad,iha,iss,sat,senB | IncFIA,IncI1,IncFIB(AP001918) | ColRNAI, Col(MG828),Col156,IncX1,IncX4,Col8282 | IncI1 [Unknown ST], IncF[F-:A2:B20] |
| ST-131-HVR2496 | blaTEM-1B | gad,iha,iss,sat,senB | IncFII(pRSB107),IncFIA,IncFIB(AP001918) | Col8282,Col156,ColpVC | IncF[F1:A2:B20] |
| ST-131-HVM834 | aac(6')Ib-cr,aadA5,blaCTX-M-15,blaOXA-1,blaTEM-1A,catB3-like,dfrA17,mph(A),sul1,tet(A) | gad,iha,iss,nfaE,sat | IncFII(pRSB107),IncFII,IncI1,IncFIA | ColRNAI, Col(MG828), Col156,IncX1,IncX4,Col8282 | IncI1[Unknown ST], IncF[F2:A1:B-] |
| ST-131-HVM826 | blaTEM-1A | gad,iha,sat | IncFII(pRSB107),IncFIA,IncFIB(AP001918),IncFII(pCoo) | Col156 | IncF[F1:A2:B20] |
| ST-131-HVM52 | blaTEM-1C,tet(A)-like | cnf1,gad,iroN,iss,mchF | IncFII(pRSB107),IncFII,IncI1,IncFIB(AP001918) | IncB/O/K/Z, ColRNAI | IncF[F2:A-:B1], IncI1[Unknown ST] |
| ST-131-HVM5 | | gad,iha,iss,sat,senB | | Col8282,ColRNAI,Col156, Col(MG828) | NA |
| ST-131-HVM3189 | aac(3)-IId-like,aadA5,blaTEM-1B,mph(A),strA,strB-like,sul1,sul2,tet(A) | gad,iha,iss,sat,senB | IncFII(pRSB107),IncFIA,IncFIB(AP001918) | Col8282,Col156,Col(MG828) | IncF[F1:A2:B20] |
| ST-131-HVM3017 | aac(6')Ib-cr,blaCTX-M-15,blaOXA-1,catB3-like,tet(A) | cnf1,gad,iha,iss,sat | IncFII,IncFIB(AP001918),IncFIA | | IncF [F36:A20:B1] |
| ST-131-HVM277 | blaTEM-1C,tet(A) | astA,cnf1,gad,iroN,iss,mchF | IncFII,IncFIB(AP001918) | ColRNAI,Col(MG828) | IncF[F2:A-:B1] |
| ST-131-HVM2757 | aadA1,aadA2,blaTEM-1B,cmlA1-like,dfrA12,sul3,tet(B) | gad,ireA,iroN,iss,mchF,vat | IncFIA(HI1),IncHI1B(R27),IncFIC(FII),IncFIB(AP001918),IncHI1A | p0111,ColRNAI, ColpVC, Col(MG828) | IncHI1[Unknown ST],IncF[F18:A8:B1] |
| ST-131-HVM2289 | aadA1,aadA2,blaTEM-1C,cmlA1-like,dfrA12,sul3,tet(A) | ccl,cnf1,gad, iroN,iss,mchF | IncFII,IncI1,IncFIB(AP001918) | ColRNAI | IncI1[ST-48], IncF[F2:A-:B1] |
| ST-131-HVM2049 | | gad,ireA,iroN,iss,mchF,vat | IncFII,IncFIB(AP001918) | Col156,ColRNAI | IncF[F24:A-:B1*] |
| ST-131-HVM2044 | aadA1,aadA2,blaTEM- | ccl,cnf1,gad,iroN,iss,mchF | IncFII,IncI1,IncFIB(AP001918) | ColRNAI | IncF[F2:A-:B1],IncI1[ST- |

| | | | | | |
|---|---|---|---|---|---|
| | 1C,cmlA1-like,dfrA12,sul3,tet(A) | | | | 48] |
| ST-131-HVM1997 | aac(6')Ib-cr,aadA5,blaCTX-M-15,blaOXA-1,catB3-like,dfrA17,mph(A),sul1,tet(A) | gad,iha,iss,nfaE,sat | IncFII,IncFIA | Col8282,Col156,ColRNAI | IncF[F2:A1:B-] |
| ST-131-HVM1619 | aac(6')Ib-cr,aadA5,blaCTX-M-15,blaOXA-1,catB3-like,dfrA17,mph(A),sul1,tet(A) | gad,iha,iss,nfaE | IncFII,IncFIA | Col8282,ColRNAI,Col156,IncX4,Col(MG828) | IncF[F2:A1:B-] |
| ST-131-HVM1299 | aac(6')Ib-cr,blaCTX-M-15,blaOXA-1,catB3-like,tet(A) | cnf1,gad,iha,iss,sat | IncFIA,IncFIB(AP001918),IncFII | | IncF [F36:A20:B1] |
| ST-131-HVM1181 | | gad,ireA,iroN,iss,mchF,vat | IncFII,IncFIB(AP001918) | ColRNAI | IncF[F24:A-:B1*] |
| ST-131-HVM1147 | | gad,iha,iss,nfaE,sat,senB | IncFII(29),IncFIB(AP001918) | Col156 | IncF[F29:A-:B10] |
| ST-131-B36EC | aac(6')Ib-cr,aadA5,blaCTX-M-15,blaOXA-1,catB3-like,dfrA17,mph(A),sul1,tet(A) | gad,iha,iss,nfaE,sat | IncFII,IncFIA | Col(MG828) | IncF[F2:A1:B-] |
| S_sonnei_Ss046 | aadA1,dfrA1,strA,strB,sul2,tet(A)-like | capU,gad,ipaD,ipaH9.8,lpfA,senB,sigA,virF | IncFII | Col(BS512),Col156,ColRNAI | IncF[F27:A-:B-] |
| S_flexneri_2a_str_301 | | capU,gad,ipaD,ipaH9.8,lpfA,pic,sepA,sigA,virF | IncFII | | IncF[F27:A-:B-] |
| S_dysenteriae_Sd197 | | capU,gad,ipaD,ipaH9.8,iroN,stx1A,stx1B | IncFII | ColE10 | IncF[F25:A-:B-] |
| S_boydii_Sb227 | | capU,gad,iha,ipaH9.8,senB,sigA,virF | IncFII | | IncF[F27:A-:B-] |
| E_coli_UTI89 | | cnf1,gad,iroN,iss,senB,sfaS,vat | IncFII(29),IncFIB(AP001918) | Col156 | IncF[F29:A-:B10] |
| E_coli_UMN026 | aadA5,blaTEM-1B,catA1-like,dfrA17,mph(A),sul1,tet(B) | air,eilA,gad,iha,iss,lpfA,sat,senB | IncFII(29),IncFIB(AP001918) | IncX1,Col156 | IncF[F29:A-:B10] |
| E_coli_str_K-12_substr_MG1655 | | gad,iss | | | NA |
| E_coli_SMS-3-5 | aadA2,aph(3')-Ia,blaTEM-1B,catA2-like,dfrA14-like,strA-like,strB-like,sul2,tet(A) | air,astA,cma,eilA,gad,iha,lpfA | IncFII,IncFIB(AP001918) | Col8282, ColRNAI | IncF[F24:A-:B1] |
| E_coli_SE11 | tet(C)-like | gad,iss,lpfA | IncFII(pSE11),IncI1,IncFIB(AP001918) | Col8282,ColRNAI | IncI1[ST-14],IncF[F20:A-:B12] |
| E_coli_S88 | | gad,ireA,iroN,iss | IncFII,IncFIB(AP | | IncF[F24:A-:B1] |

| | | | | | |
|---|---|---|---|---|---|
| | | ,mchF,vat | 001918) | | |
| E_coli_O83-H1_str_NRG_857C | aadA1,blaTEM-1B-like,catA1-like,dfrA1,mph(B),strA,strB,sul1,sul2,tet(A) | cma,gad,iroN,iss,vat | IncFIC(FII),IncFIB(AP001918) | IncQ1 | IncF[F18:A-:B1] |
| E_coli_O7-K1_str_CE10 | | air,capU,eilA,gad,iha,iss,lpfA,sat,senB | IncFII(29),IncFIB(AP001918) | Col(MG828),Col156,ColRNAI | IncF[F29:A-:B10] |
| E_coli_O55-H7_str_RM12579 | blaTEM-1B,strA,strB,sul2 | astA,eae,efa1,espA,espB,espF,espJ,etpD,gad,iss,nleA,nleB,nleC,tccP,tir | IncFIB(AP001918) | p0111,ColRNAI | IncF[F-:A-:B15] |
| E_coli_O157-H7_str_EDL933 | | astA,eae,ehxA,espA,espB,espF,espJ,espP,etpD,gad,iha,iss,katP,nleA,nleB,nleC,stx1A,stx1B,stx2A,stx2B,tccP,tir,toxB | IncFII,IncFIB(AP001918) | | IncF[F23:A-:B3] |
| E_coli_O127-H6_str_E2348-69 | strA,strB,sul2 | astA,bfpA,cif,eae,efa1,espA,espC,espF,espJ,gad,nleA,nleB,nleC,perA,tir | IncFIB(pB171),IncFII(pCoo) | ColRNAI | IncF[F13:A-:B23] |
| E_coli_O104-H4_str_2011C-3493 | blaCTX-M-15,blaTEM-1B,dfrA7,strA,strB,sul1-like,sul2,tet(A) | aaiC,aap,aar,aatA,aggA,aggB,aggC,aggD,aggR,capU,gad,iha,lpfA,mchB,mchC,mchF,ORF3,ORF4,pic,sepA,sigA,stx2A,stx2B | IncFII(pRSB107),IncI1,IncFIB(AP001918) | IncQ1,Col(MG828) | IncI1[ST-31],IncF[F48:A-:B25] |
| E_coli_Nissle_1917 | | gad,iha,iroN,iss,mchB,mchC,mchF,mcmA,pic,sat,vat | | Col(MGD2),ColRNAI | NA |
| E_coli_M4163 | | capU,gad,ipaD,senB,virF | IncFII | Col(MG828) | IncF[F28:A-:B-] |
| E_coli_LF82 | | gad,iss,vat | | | NA |
| E_coli_IAI39 | | air,capU,eilA,gad,lpfA | | | NA |
| E_coli_HS | | gad | | | NA |
| E_coli_ETEC_H10407 | | astA,cfaC,eatA,gad,ltcA,sta1 | IncFII(29),IncFII | Col(MGD2) | IncF[F12:A-:B-] |
| E_coli_E24377A | strA,strB,sul2 | astA,capU,eatA,gad,lpfA,ltcA | IncI1,IncFII(pHN7A8),IncFII(pCoo) | ColRNAI | IncI1[Unknown ST],IncF[F11:A-:B-] |
| E_coli_CFT073 | | gad,iha,ireA,iroN,iss,mchB,mchC,mchF,mcmA,pic,sat,vat | | | NA |
| E_coli_55989 | tet(B) | aaiC,capU,gad,iha,lpfA,pic,sigA | | | NA |
| E_coli_4608-58 | | capU,gad,iha,ipaD,senB,sigA,virF | IncFII | | IncF[F28:A-:B-] |
| E_coli_042 | aadA1,catA1-like,sul1,tet(A) | aafA,aafB,aafC,aafD,aaiC,aap,aar,aatA,aggR,air,a | IncFIC(FII) | | IncF[F9:A-:B-] |

| | | | | | |
|---|---|---|---|---|---|
| | | stA,capU,eilA,gad,lpfA,mchB,mchC,mchF,mcmA,ORF3,ORF4,pet,pic | | | |
